# Clinicopathological discordance in biopsy-proven nephrosclerosis: a nationwide cross-sectional study of the Japan Renal Biopsy Registry (J-RBR)

**DOI:** 10.1007/s10157-021-02161-1

**Published:** 2021-11-23

**Authors:** Keiichi Sumida, Asami Takeda, Kengo Furuichi, Noriko Uesugi, Yoshifumi Ubara, Hiroshi Sato, Hitoshi Sugiyama, Akira Shimizu, Hitoshi Yokoyama

**Affiliations:** 1 Division of Nephrology, Department of Medicine, University of Tennessee Health Science Center, 956 Court Ave., Suite A220, Memphis, TN 38163 USA; 2 Kidney Disease Center, Japanese Red Cross Nagoya Daini Hospital, Nagoya, Japan; 3 Department of Nephrology, Kanazawa Medical University School of Medicine, Ishikawa, Japan; 4 Department of Pathology, Faculty of Medicine, Fukuoka University, Fukuoka, Japan; 5 Nephrology Center, Toranomon Hospital, Tokyo, Japan; 6 Department of Internal Medicine, Sendai Hospital of East Japan Railway Company, Sendai, Japan; 7 Department of Human Resource Development of Dialysis Therapy for Kidney Disease, Okayama University Institute of Academic and Research of Medicine, Dentistry and Pharmaceutical Sciences, Okayama, Japan; 8 Department of Analytic Human Pathology, Nippon Medical School, Tokyo, Japan

**Keywords:** Clinical diagnosis, Discordance, Japan Renal Biopsy Registry (J-RBR), Nephrosclerosis, Pathological diagnosis, Renal biopsy

## Abstract

**Background:**

Patients with nephrosclerosis display heterogenous clinical phenotypes, often leading to a clinical diagnosis discordant with pathological nephrosclerosis diagnosis. However, little is known about clinical factors associated with clinicopathological discordance of biopsy-proven nephrosclerosis.

**Methods:**

In a cross-sectional study of 891 patients with biopsy-proven nephrosclerosis registered in the Japan Renal Biopsy Registry (J-RBR) between July 2007 and June 2016, we examined clinical characteristics associated with a pre-biopsy clinical diagnosis discordant with pathological nephrosclerosis diagnosis using multivariable logistic regression with adjustment for relevant clinical characteristics.

**Results:**

Overall, the mean (SD) age was 58.6 (13.7) years; 67.6% of patients were male; and 63.2% were on antihypertensive drugs. The median estimated glomerular filtration rate (eGFR) was 43.8 mL/min/1.73 m^2^ and the median proteinuria was 0.5 g/day. Of the 891 patients, 497 (55.8%) had a clinical diagnosis discordant with pathological nephrosclerosis diagnosis, with chronic nephritic syndrome being the most common (> 75%) discordant clinical diagnosis. After multivariable adjustment, age (odds ratio 1.34, [95% confidence interval, 1.16–1.55], per 10 years increase), eGFR (1.10 [1.00–1.21], per 10 mL/min/1.73 m^2^ increase), and proteinuria (1.20 [1.03–2.16], per 1 g/day decrease) were found to be significantly associated with the clinicopathological discordance.

**Conclusions:**

Patients with older age, higher eGFR, and lower proteinuria had significantly higher likelihood of being clinically diagnosed with other glomerular disease in patients with biopsy-proven nephrosclerosis. Our findings highlight the heterogeneous clinical phenotypes of nephrosclerosis and suggest the need for continuous improvement of clinical diagnostic accuracy as well as for wider kidney biopsy indications for nephrosclerosis.

**Supplementary Information:**

The online version contains supplementary material available at 10.1007/s10157-021-02161-1.

## Introduction

Nephrosclerosis is one of the leading causes of end-stage renal disease (ESRD), accounting for ~ 15% of all incident ESRD cases in Japan and even higher (~ 28%) in the United States, and these numbers have been steadily increasing along with the increasing prevalence of hypertension across the world [1, 2]. Despite this fact, the diagnosis of nephrosclerosis is often one of exclusion and typically based on clinical manifestations, such as long-standing hypertension, the absence of diabetes, hematuria, and overt proteinuria, and the absence of other known CKD causes [3]; and hence the diagnostic accuracy of nephrosclerosis has long been questioned, as reflected by the wide range of reported incidence of ESRD due to nephrosclerosis across studies [3].

With accumulating evidence of the prognostic impact of nephrosclerosis on kidney and cardiovascular outcomes [4–7], the accurate diagnosis of nephrosclerosis has become more important than ever. Although efforts have been made to develop clinical nephrosclerosis criteria (e.g., history of hypertension and low proteinuria) [3], their diagnostic accuracy remains a topic of deliberation, presumably due to the variety of etiologies which make it difficult to uniformly characterize the clinical phenotype of nephrosclerosis. In fact, it is common in clinical practice to encounter cases suspected to have a glomerular disease (and hence indicated for renal biopsy) but end up with a pathological diagnosis with nephrosclerosis. Similarly, it is not very rare to see patients who were clinically diagnosed with nephrosclerosis but had a pathological diagnosis of other glomerulopathy, although only a limited number of patients with clinical diagnosis of nephrosclerosis undergo kidney biopsy.

Given the limited diagnostic accuracy of the current clinical criteria and the relatively high harm-to-benefit ratio of performing renal biopsy to diagnose nephrosclerosis [3], continuous efforts are needed to fill in the gap between clinical and pathological diagnoses of nephrosclerosis. Toward this end, it is essential to understand the potential heterogeneity of clinical characteristics of patients with nephrosclerosis that leads to the discordance between clinical and pathological diagnosis of nephrosclerosis. Currently, however, there are scarce studies investigating the factors associated with the clinicopathological discordance in nephrosclerosis, presumably due to the lack of information of pre-biopsy clinical diagnosis in large renal biopsy databases. In the present study, using a unique and large nationwide multicenter renal biopsy registry in Japan, where pre-biopsy clinical diagnoses are available, we aimed to identify clinical factors associated with a (pre-biopsy) clinical diagnosis discordant with (post-biopsy) pathological nephrosclerosis diagnosis in patients with biopsy-proven nephrosclerosis.

## Methods

### Study design and study population

This is a cross-sectional study of de-identified clinical data obtained from the Japan Renal Biopsy Registry (J-RBR), which is a nationwide, web-based, multicenter prospective registry of renal biopsies in Japan. J-RBR was organized by the Committee for the Standardization of Renal Pathological Diagnosis and the Working Group for the Renal Biopsy Database of the Japanese Society of Nephrology (JSN) in 2007. J-RBR is registered under the Clinical Trial Registry of UMIN (registration number, UMIN000000618), and the study protocol was approved by the Ethics Committee of the JSN in accordance with the Declaration of Helsinki (approval number 51). Written informed consent was obtained from all patients who participated in this registry. Details of the J-RBR have been published previously [8].

In the present study, among 31,409 patients with biopsy-proven renal disease who were registered in the J-RBR between July 2007 and June 2016, we identified 1068 patients who had a pathological diagnosis of hypertensive nephrosclerosis (as classified by pathogenesis) and either nephrosclerosis or sclerosing glomerulonephritis (as classified by histopathology; see below), irrespective of their clinical diagnosis. Since it is not rare in clinical practice to see young adults diagnosed with nephrosclerosis, we included patients ≥ 20 years in this study. After excluding patients who were < 20 years (*n* = 5) and had history of diabetes (*n* = 172), 891 patients were included in our final cohort.

### Clinical data collection

Baseline clinical data available in the J-RBR database were collected at the time of renal biopsy. These include patient demographic and anthropometric characteristics (age, sex, height, body weight, and body mass index [BMI]), blood pressure measurements, use of antihypertensive drugs, medical history of hypertension and diabetes, laboratory measures (serum total protein, serum albumin, total cholesterol, hemoglobin A1c, serum creatinine, estimated glomerular filtration rate [eGFR], proteinuria assessed by 24-h urine protein excretion, urine protein–creatinine ratio, and dipstick protein, and hematuria assessed by sediment red blood cells [RBCs] and dipstick hematuria), and clinical and pathological diagnoses. eGFR was calculated using the modified equation for Japanese individuals [9].

Clinical diagnosis was determined by primary nephrologists primarily based on clinical manifestations and classified in the registry as follows: acute nephritic syndrome, rapidly progressive nephritic syndrome, recurrent or persistent hematuria, chronic nephritic syndrome, nephrotic syndrome, renal disorder with metabolic disease, renal disorder with collagen disease or vasculitis, hypertensive nephropathy, inherited renal disease, acute renal failure, drug-induced nephropathy, renal transplantation, and others. Pathological diagnosis was classified according to the pathogenesis (e.g., lupus nephritis) and histopathology (e.g., endocapillary proliferative glomerulonephritis). More detailed information about the definition of each clinical diagnosis and the classification of pathological diagnosis has been provided elsewhere [8]. In the present study, the pathological nephrosclerosis diagnosis was defined as having a pathological diagnosis of hypertensive nephrosclerosis (by pathogenesis) and either nephrosclerosis or sclerosing glomerulonephritis (by histopathology).

### Statistical analysis

Baseline patient characteristics were presented as number (percentages) for categorical variables and mean (standard deviation [SD]) for continuous variables with a normal distribution or median (interquartile interval [IQI]) for those with a skewed distribution. The distributions of key clinical characteristics, including age, systolic blood pressure, eGFR, and 24-h urine protein, were visually depicted in histograms. According to the concordance and discordance of clinical diagnosis (i.e., hypertensive nephropathy vs. others) with pathological nephrosclerosis diagnosis, patients were classified into two groups. Differences between the groups were assessed using the chi-squared test, unpaired *t* test, or Wilcoxon rank-sum test, as appropriate. To identify factors independently associated with the clinicopathological discordance of nephrosclerosis, the associations between baseline clinical characteristics and discordance of clinical diagnosis (i.e., glomerular disease other than hypertensive nephropathy) were examined using univariable and multivariable logistic regression models. Based on theoretical consideration and data availability, the following explanatory variables were included simultaneously in a single multivariable model: age, sex, BMI, systolic blood pressure, the use of antihypertensive drugs, serum albumin, total cholesterol, eGFR, 24-h urine protein, and hematuria at baseline. Since the presence of hematuria is a key component in considering indications for renal biopsy, we conducted a stratified analysis by the presence or absence of hematuria (≥ 5 vs. < 5 sediment RBCs/HPF) using the multivariable-adjusted model. Of the variables included in the multivariable model, data points were missing for BMI (1.2%), systolic blood pressure (16.3%), antihypertensive use (16.2%), serum albumin (1.9%), total cholesterol (3.6%), eGFR (0.1%), 24-h urine protein (24.1%), and hematuria (1.7%). Of the 891 patients, 527 (59.1%) had complete data available for multivariable-adjusted analyses. Missing values were not imputed in the primary analysis, but were substituted using multiple imputation procedures in a sensitivity analysis.

A threshold of statistical significance was set at the level of *P* < 0.05 for all analyses. Statistical analyses were conducted in STATA/MP Version 16 (STATA Corporation, College Station, TX).

## Results

### Baseline characteristics

**Fig. 1:**
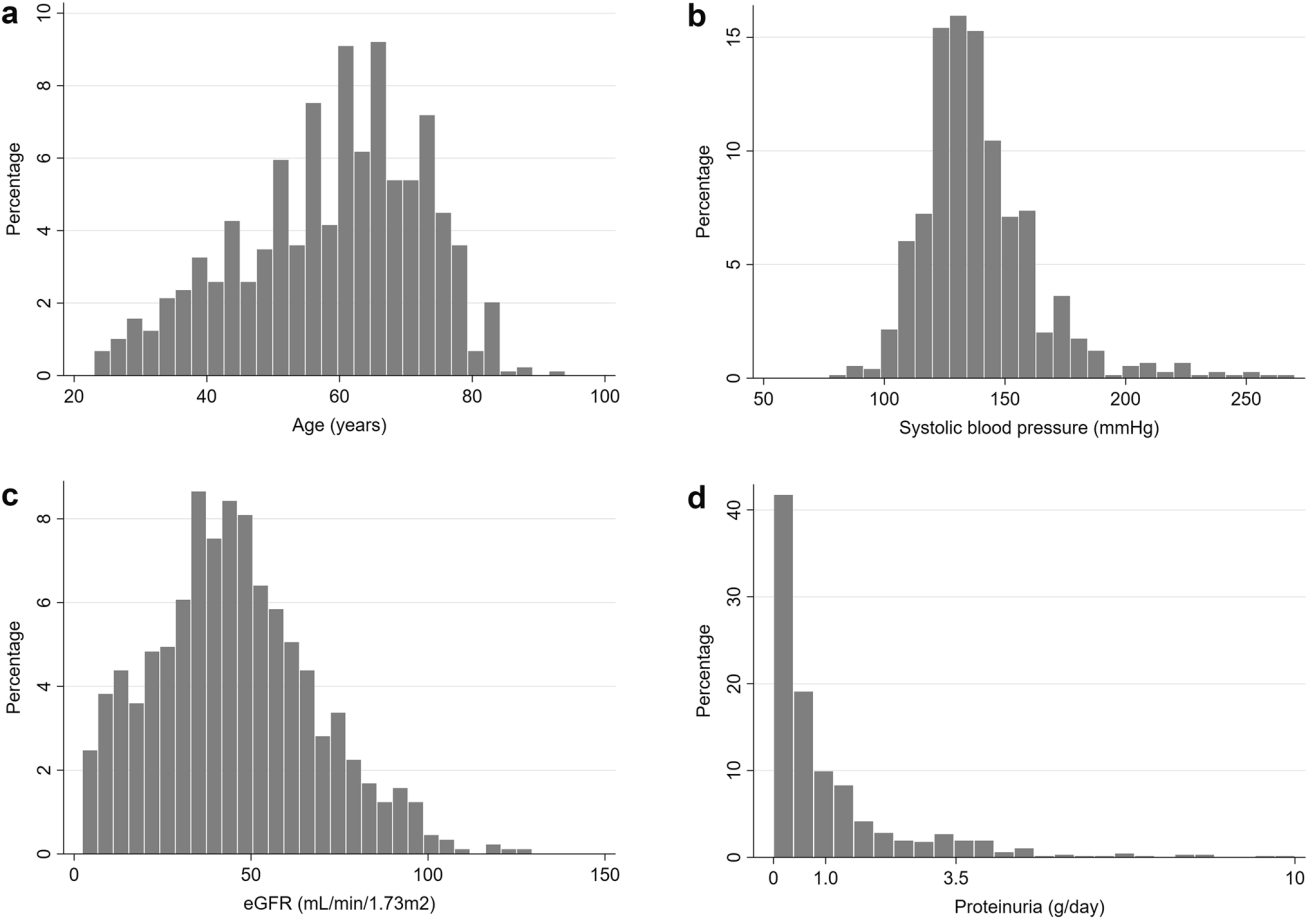
Distributions of key clinical characteristics in patients with biopsy-proven nephrosclerosis. **a** Age, **b** systolic blood pressure, **c** eGFR, and **d** proteinuria. *eGFR* estimated glomerular filtration rate

Patients’ baseline characteristics in the overall cohort and stratified by clinical diagnostic status (i.e., concordant vs. discordant with pathological nephrosclerosis diagnosis) are presented in Table 1. Overall, the mean age was 58.6 ± 13.7 years; 67.6% of patients were male; and 63.2% were on antihypertensive drugs. The median (IQI) eGFR was 43.8 (29.5–59.2) mL/min/1.73 m^2^ and the median (IQI) proteinuria was 0.5 (0.2–1.3) g/day. The overall distributions of key clinical characteristics (i.e., age, systolic blood pressure, eGFR, and proteinuria) are presented in Fig. 1. Of the 891 patients, 497 (55.8%) had a clinical diagnosis discordant with pathological nephrosclerosis diagnosis. Compared with patients with concordant clinicopathological diagnosis, those with discordant clinicopathological diagnosis were older and less likely to be male, had lower systolic and diastolic blood pressure, and were less likely to use antihypertensive drugs. They also had higher eGFR and lower proteinuria levels, and greater prevalence of hematuria (≥ 5 sediment RBCs/HPFs) at the time of renal biopsy.

**Table 1:** Baseline patient characteristics overall and according to clinicopathological diagnosis of biopsy-proven nephrosclerosis

Characteristics	Total (*n* = 891)	Clinicopathological diagnosis		*P* value*
		Concordant (n = 394)	Discordant (*n* = 497)	
Age (years)	58.6 ± 13.7	55.8 ± 14.6	60.8 ± 12.5	< 0.001
Male sex	602 (67.6)	282 (71.6)	320 (64.4)	0.023
Height (cm)	162.6 ± 9.3	163.6 ± 9.1	161.7 ± 9.5	0.003
Body weight (kg)	65.3 ± 13.8	66.6 ± 14.6	64.2 ± 13.1	0.014
Body mass index	24.5 ± 4.0	24.7 ± 4.2	24.4 ± 3.8	0.28
Systolic blood pressure (mmHg)	139.1 ± 24.6	143.4 ± 28.7	135.7 ± 20.1	< 0.001
Diastolic blood pressure (mmHg)	82.6 ± 17.6	85.2 ± 20.8	80.6 ± 14.2	< 0.001
Antihypertensive use	563 (63.2)	267 (67.8)	296 (59.6)	0.025
Laboratory parameters				
Serum total protein (g/dL)	7.0 ± 0.7	7.0 ± 0.8	7.0 ± 0.7	0.97
Serum albumin (g/dL)	4.0 ± 0.6	4.0 ± 0.6	4.0 ± 0.6	0.97
Total cholesterol (mg/dL)	203.5 ± 46.2	207.8 ± 46.5	200.1 ± 45.8	0.015
Serum creatinine (mg/dL)	1.3 [0.9, 1.8]	1.4 [1.0, 2.2]	1.2 [0.9, 1.6]	< 0.001
eGFR (mL/min/1.73 m^2^)	43.8 [29.5, 59.2]	40.6 [25.5, 57.3]	46.3 [33.4, 61.3]	< 0.001
eGFR categories (mL/min/1.73 m^2^)				< 0.001
≥ 90	37 (4.2)	17 (4.3)	20 (4.0)	
60 to < 90	175 (19.6)	63 (16.0)	112 (22.5)	
30 to < 60	446 (50.0)	185 (47.0)	261 (52.5)	
15 to < 30	137 (15.4)	65 (16.5)	72 (14.5)	
< 15	95 (10.7)	63 (16.0)	32 (6.5)	
Unknown	1 (0.1)	1 (0.2)	0 (0)	
Proteinuria (g/day)	0.5 [0.2, 1.3]	0.8 [0.3, 1.6]	0.4 [0.1, 1.1]	< 0.001
Urine PCR (g/gCr)	0.9 [0.3, 2.0]	0.9 [0.3, 2.0]	0.8 [0.3, 2.0]	0.29
Urine dipstick protein categories				0.004
Negative	150 (16.8)	49 (12.4)	101 (20.3)	
Trace	95 (10.7)	45 (11.4)	50 (10.1)	
1 +	187 (21.0)	85 (21.6)	102 (20.5)	
2 +	287 (32.2)	122 (31.0)	165 (33.2)	
≥ 3 +	172 (19.3)	93 (23.6)	79 (15.9)	
Sediment RBC categories (/HPF)				0.006
< 5	623 (69.9)	297 (75.4)	326 (65.6)	
5 to < 10	103 (11.6)	32 (8.1)	71 (14.3)	
10 to < 30	97 (10.9)	36 (9.1)	61 (12.3)	
≥ 30	68 (7.6)	29 (7.4)	39 (7.8)	

Data are presented as number (percentage), mean ± standard deviation, or median [interquartile range]

*eGFR* estimated glomerular filtration rate, *HPF* high power field, *PCR* protein-to-creatinine ratio, *RBC* red blood cell

*Comparing differences between concordant and discordant cases

### Pre-biopsy clinical diagnosis discordant with pathological nephrosclerosis diagnosis

Table 2 shows pre-biopsy clinical diagnoses in 497 patients who were clinically suspected to have glomerular disease other than hypertensive nephrosclerosis but were pathologically diagnosed with nephrosclerosis. Among these, the most common clinical diagnosis was chronic nephritic syndrome in 377 patients (75.9%), followed by nephrotic syndrome (4.8%), rapidly progressive nephritic syndrome (3.2%), acute renal failure (2.0%), recurrent or persistent hematuria (1.8%), renal disorder with collagen disease or vasculitis (1.8%), renal disorder with metabolic syndrome (1.6%), acute nephritic syndrome (0.6%), drug-induced nephropathy (0.4%), and others (7.9%).

**Table 2:** Pre-biopsy clinical diagnosis other than hypertensive nephropathy in patients with biopsy-proven nephrosclerosis (*n* = 497)

Clinical diagnosis	*n* (%)
Chronic nephritic syndrome	377 (75.9)
Nephrotic syndrome	24 (4.8)
Rapidly progressive nephritic syndrome	16 (3.2)
Acute renal failure	10 (2.0)
Recurrent or persistent hematuria	9 (1.8)
Renal disorder with collagen disease or vasculitis	9 (1.8)
Renal disorder with metabolic syndrome	8 (1.6)
Acute nephritic syndrome	3 (0.6)
Drug-induced nephropathy	2 (0.4)
Others	39 (7.9)

### Clinical characteristics associated with clinicopathological discordance

Table 3 shows the associations between baseline clinical characteristics and clinicopathological discordance using univariable and multivariable-adjusted logistic regression analyses. In the univariable model, all examined characteristics except BMI were significantly associated with clinicopathological discordance. After multivariable adjustment, age (OR 1.34, [95% confidence interval (CI) 1.16–1.55], per 10 year increase), eGFR (OR 1.10 [95% CI 1.00–1.21], per 10 mL/min/1.73 m^2^ increase), and proteinuria (OR 1.20 [95% CI 1.03–2.16], per 1 g/day decrease) were found to be significantly associated with clinicopathological discordance (Table 3).

**Table 3:** Associations of clinical characteristics with clinicopathological discordance in biopsy-proven nephrosclerosis

Characteristics	Univariable			Multivariable-adjusted		
	Odds ratio	95% CI	*P* value	Odds ratio	95% CI	*P* value
Age (10 years higher)	1.31	1.19–1.45	< 0.001	1.34	1.16–1.55	< 0.001
Male sex (vs. female)	0.72	0.54–0.96	0.023	0.74	0.48–1.12	0.15
BMI (1 unit higher)	0.98	0.95–1.02	0.28	0.99	0.94–1.04	0.67
Systolic BP (10 mmHg higher)	0.88	0.82–0.93	< 0.001	0.96	0.88–1.05	0.41
Antihypertensive use (vs. non-use)	1.01	1.00–1.01	0.027	1.01	0.99–1.02	0.10
Serum albumin (1 g/dL higher)	1.00	0.79–1.28	0.04	0.90	0.61–1.32	0.58
Total cholesterol (10 mg/dL higher)	0.96	0.94–0.99	0.016	0.97	0.93–1.02	0.26
eGFR (10 mL/min/1.73 m^2^ higher)	1.10	1.04–1.17	0.002	1.10	1.00–1.21	0.046
Proteinuria (1 g/day lower)	1.20	1.07–1.34	0.001	1.20	1.03–2.16	0.017
Hematuria (≥ 5 [vs. < 5] sediment RBCs/HPF)	1.61	1.20–2.16	0.002	1.42	0.94–2.16	0.097

*BMI* body mass index, *BP* blood pressure, *CI* confidence interval, *eGFR* estimated glomerular filtration rate, *HPF* high-power field, *RBCs* red blood cells

When the associations were examined in a subgroup of patients with and without hematuria, age (OR 1.41 [95% CI 1.19–1.67], per 10 year increase) and proteinuria (OR 1.23 [95% CI 1.03–1.48], per 1 g/day decrease) remained statistically significant among those without hematuria; while among those with hematuria, eGFR was the only factor significantly associated with clinicopathological discordance (OR 1.21 [95% CI 1.02–1.45], per 10 mL/min/1.73 m^2^ increase; Supplementary Table 1). Results were largely similar even after imputing missing data in the primary multivariable analysis, with an exception that hematuria was additionally identified as a factor independently associated with clinicopathological discordance (OR 1.58 [95% CI 1.16–2.16], for ≥ 5 [vs. < 5] sediment RBCs/HPF; Supplementary Table 2).

## Discussion

In this large nationwide multicenter renal biopsy registry in Japan, we found that more than half (55.8%) of the patients with biopsy-proven nephrosclerosis were clinically diagnosed with other glomerular disease, most commonly (~ 75%) with chronic nephritic syndrome. We also demonstrated that patients with biopsy-proven nephrosclerosis who were older (per 10 years), had higher eGFR (per 10 mL/min/1.73 m^2^), and had lower proteinuria (per 1 g/day) were at ~ 1.3-fold, ~ 1.1-fold, and ~ 1.2-fold greater likelihood of being clinically diagnosed with other glomerular disease, respectively, independent of other relevant clinical characteristics.

Over the past few decades, considerable efforts have been made to improve clinical nephrosclerosis criteria, defined based primarily on history of hypertension and the absence of diabetes, hematuria, overt proteinuria, and other known CKD causes [3]. Several studies have reported the diagnostic accuracy of these clinical nephrosclerosis criteria [10–15], with their reported positive predictive values ranging up to 97% [12], suggesting their high accuracy and clinical applicability for the diagnosis of nephrosclerosis. However, all of these previous studies had relatively small sample sizes (< 100 patients) and often had suboptimal study designs, which can lead to substantial selection bias and inaccuracy of reported predictive values. In fact, a recent study from a large Norwegian Kidney Biopsy Registry demonstrated that the current clinical nephrosclerosis criteria had very low sensitivity (17%) but high specificity (94%) for making an accurate diagnosis of nephrosclerosis [16]. The same study also revealed that the pathological diagnosis of nephrosclerosis, defined as the presence of histopathological arterionephrosclerosis, was significantly associated with older age, male sex, not having diabetes, higher blood pressure, lower proteinuria, and not having hematuria [16]. Importantly, however, these associations were examined by comparing clinical characteristics between patients with and without biopsy-verified nephrosclerosis (i.e., the presence of histopathological arterionephrosclerosis); and hence, despite the detailed examination of clinical indications for renal biopsy, factors associated with clinicopathological discordance only among patients with biopsy-proven nephrosclerosis remain unclear.

In this context, the availability of pre-biopsy clinical diagnosis in our study would be of particular value, allowing the identification of clinical factors affecting the decision-making of clinical diagnosis in patients with biopsy-proven nephrosclerosis, which in turn could provide novel insights into underlying etiologies and clinical phenotype of nephrosclerosis. More specifically, for example, our results showing the significant association of older age with a higher likelihood of having a discordant clinicopathological diagnosis may reinforce the importance of aging process in the development and progression of histological abnormalities related to nephrosclerosis [6]. Given the fact that nephrosclerosis is progressing against hypertension and aging and is increasing over time, our findings may lead to the emergence of more and more discrepancies between clinical and pathological diagnoses of nephrosclerosis. Furthermore, together with the finding for lower proteinuria that was independently associated with clinicopathological discordance only in patients without hematuria, the significant association for older age observed only in those without hematuria suggests that the histological abnormalities related to aging-related nephrosclerosis may develop without accompanying overt proteinuria and hematuria. Nonetheless, it is important to note that nephrosclerosis may present with server proteinuria despite its similar pathological picture to that without proteinuria [17], potentially through different mechanisms involved in the pathogenesis. Similarly, the significant association for higher eGFR that was evident only in patients with hematuria may suggest the existence of distinct pathogenic mechanisms underlying nephrosclerosis, which could manifest predominantly as hematuria and preserved kidney function. The certain prevalence of hematuria (~ 20% to 40%) [5, 6, 18] reported across studies of patients with biopsy-proven nephrosclerosis may also support this observation. However, perhaps, most importantly, our results highlighting various clinical features in patients with biopsy-proven nephrosclerosis will help raise awareness of heterogeneous clinical phenotypes of nephrosclerosis and also aid clinicians in making an accurate diagnosis of nephrosclerosis by balancing harm-to-benefit ratio of performing renal biopsy, which could consequently lead to improved management strategies (e.g., by clinical phenotypes) and better clinical outcomes in patients with nephrosclerosis.

Despite the substantial strengths of our study including its large sample size and availability of pre-biopsy clinical diagnosis, the study results must be interpreted in light of several limitations. Our study population was limited to those who had undergone kidney biopsy with certain clinical indications and hence may not be representative of nephrosclerosis patients. Data of immunofluorescence and electron microscopy studies are not available in the J-RBR. Although we excluded patients with immunological or histological evidence of other glomerular disease or those with history of diabetes, we cannot eliminate the possibility of misclassification of patients with pathological nephrosclerosis diagnosis, due in part to the manual registration process that is prone to human error. In addition, the diagnostic criteria of nephrosclerosis might not be uniform among the pathologists. Similar misclassification is also possible for clinical diagnoses in this study. Our study lacked detailed pathological information, such as degrees of glomerular ischemic changes and interstitial fibrosis, and therefore, the associations of individual pathological findings of nephrosclerosis with the clinicopathological discordance were not evaluated. Finally, due to the limited clinical data available in the J-RBR, we cannot exclude the possibility of unmeasured clinical factors (e.g., serum uric acid, uric acid and lipid lowering agents, left ventricular hypertrophy and lifestyle factors, such as smoking) that might have affected the decision-making of clinical nephrosclerosis diagnosis.

In conclusion, in this nationwide study of 891 Japanese patients with biopsy-proven nephrosclerosis, we described the discordance between clinical and pathological diagnosis of nephrosclerosis and found that patients with older age, higher eGFR, and lower proteinuria had significantly higher likelihood of being clinically diagnosed with other glomerular disease in patients with biopsy-proven nephrosclerosis. Our findings highlight the heterogeneous clinical phenotypes of nephrosclerosis and suggest the need for continuous improvement of clinical diagnostic accuracy, as well as for wider kidney biopsy indications for nephrosclerosis.

## Supplementary Information

Below is the link to the electronic supplementary material.

Supplementary file1 (PDF 259 KB)
