# Supplementary material for: Clinicopathological discordance in biopsy-proven nephrosclerosis: a nationwide cross-sectional study of the Japan Renal Biopsy Registry (J-RBR)
Source: Clin Exp Nephrol. 2021 Nov 23;26(4):325–32. doi: 10.1007/s10157-021-02161-1 (PMC8930907; doi:10.1007/s10157-021-02161-1)
Supplement: Supplementary file 1 — Supplementary file1 (PDF 259 KB) [file 10157_2021_2161_MOESM1_ESM.pdf]

Supplementary Online Contents

Supplementary Table 1. Associations of clinical characteristics with clinicopathological discordance of biopsy-proven nephrosclerosis after multivariable-adjustment by hematuria subgroup

Supplementary Table 2. Associations of clinical characteristics with clinicopathological discordance of biopsy-proven nephrosclerosis after multivariable-adjustment with multiple imputations for missing data

Supplementary Appendix. The investigators and institutions participating to the Japan Renal Biopsy Registry

Supplementary Table 1. Associations of clinical characteristics with clinicopathological discordance of biopsy-proven nephrosclerosis after multivariable-adjustment by hematuria subgroup

Characteristics	No hematuria (<5 sediment RBCs/HPF)			Hematuria (≥5 sediment RBCs/HPF)		
	**Odds ratio**	**95% CI**	***P* value**	**Odds ratio**	**95% CI**	***P* value**
Age (10 years higher)	1.41	1.19-1.67	<0.001	1.17	0.85-1.60	0.33
Male sex (vs. female)	0.78	0.46-1.30	0.34	0.75	0.35-1.62	0.46
BMI (1 unit higher)	0.99	0.93-1.04	0.65	1.00	0.90-1.12	0.93
Systolic BP (10 mmHg higher)	0.97	0.88-1.08	0.64	0.97	0.84-1.14	0.73
Antihypertensive use (vs. non-use)	1.01	0.99-1.02	0.24	1.04	0.93-1.16	0.48
Serum albumin (1 g/dL higher)	0.90	0.58-1.42	0.66	0.95	0.42-2.18	0.91
Total cholesterol (10 mg/dL higher)	0.99	0.95-1.05	0.95	0.91	0.83-1.01	0.065
eGFR (10 mL/min/1.73 m^2 higher)	1.06	0.94-1.19	0.34	1.21	1.02-1.45	0.032
Proteinuria (1 g/day lower)	1.23	1.03-1.48	0.020	1.08	0.80-1.47	0.61

Note: Data were adjusted for all variables listed in this table.

Abbreviations: BMI = body mass index; BP = blood pressure; CI = confidence interval; eGFR = estimated glomerular filtration rate; HPF = high-power field; RBCs = red blood cells

Supplementary Table 2. Associations of clinical characteristics with clinicopathological discordance of biopsy-proven nephrosclerosis after multivariable-adjustment with multiple imputations for missing data

Characteristics	Odds ratio	95% CI	*P* value
Age (10 years higher)	1.27	1.14-1.42	<0.001
Male sex (vs. female)	0.81	0.59-1.10	0.18
BMI (1 unit higher)	1.02	0.98-1.06	0.26
Systolic BP (10 mmHg higher)	0.95	0.87-1.03	0.18
Antihypertensive use (vs. non-use)	1.01	0.99-1.01	0.072
Serum albumin (1 g/dL higher)	0.88	0.66-1.18	0.40
Total cholesterol (10 mg/dL higher)	0.99	0.95-1.02	0.39
eGFR (10 mL/min/1.73 m^2 higher)	1.06	0.99-1.13	0.10
Proteinuria (1 g/day lower)	1.13	1.01-1.28	0.041
Hematuria (≥5 [vs. <5] sediment RBCs/HPF)	1.58	1.16-2.16	0.004

Note: Data were adjusted for all variables listed in this table.

Abbreviations: BMI = body mass index; BP = blood pressure; CI = confidence interval; eGFR = estimated glomerular filtration rate; HPF = high-power field; RBCs = red blood cells

Supplementary Appendix. The investigators and institutions participating to the Japan Renal Biopsy Registry (J-RBR)

The following investigators and initial institutions have participated in the development of the J-RBR since 2007: Hirofumi Makino and Hitoshi Sugiyama (Okayama University), late Takashi Taguchi (Nagasaki University), Hitoshi Yokoyama (Kanazawa Medical University), Hiroshi Sato (Tohoku University), Takao Saito (Fukuoka University; present institution: Sanko Clinic) ,Yoshie Sasatomi (Fukuoka University), Yukimasa Kohda (Kumamoto University; present institution: Hikarinomori Clinic), Shinichi Nishi (Niigata University; present institution: Kobe University), Kazuhiko Tsuruya (Kyushu University), Yutaka Kiyohara (Kyushu University; present institution: Hisayama Research Institute For Lifestyle Diseases), Hideyasu Kiyomoto (Kagawa University; present institution: Tohoku Medical Megabank Organization, Tohoku University), Hiroyuki Iida (Toyama Prefectural Central Hospital; present institution: Saiseikai Takaoka Hospital), Tamaki Sasaki (Kawasaki Medical School), Makoto Higuchi (Shinshu University; present institution: National Hospital Organization Matsumoto Medical Center (Matsumoto)), Motoshi Hattori (Tokyo Women's Medical University), Kazumasa Oka (Osaka Kaisei Hospital; present institution: Hyogo Prefectural Nishinomiya Hospital), Shoji Kagami (The University of Tokushima Graduate School), Michio Nagata (University of Tsukuba), Tetsuya Kawamura (The Jikei University School of Medicine), Masataka Honda (Tokyo Metropolitan Children's Medical Center), Yuichiro Fukasawa (KKR Sapporo Medical Center ; present institution: Sapporo City General Hospital), Atsushi Fukatsu (Kyoto University Graduate School of Medicine; present institution: Fukatsu Medical Clinic), Kunio Morozumi (Japanese Red Cross Nagoya Daini Hospital; present institution: Masuko Memorial Hospital), Norishige Yoshikawa (Wakayama Medical University), Yukio Yuzawa (Fujita Health University), Seiichi Matsuo (Nagoya University Graduate School of Medicine) and Kensuke Joh (Chiba-East National Hospital; present institution: Tohoku University Graduate School of Medicine).

Hokkaido District

- Asahikawa Medical University Hospital (Division of Cardiology, Nephrology, Pulmonology and Neurology, Department of Internal Medicine), Naoyuki Hasebe, Naoki Nakagawa, Junko Chinda
- National Hospital Organization Hokkaido Medical Center (Department of Nephrology), Sekiya Shibazaki, Tomotsune Miyamoto, Masanori Ito
- Hokkaido University Graduate School of Medicine (Department of Medicine II), Saori Nishio
- Hokkaido University Graduate School of Medicine (Department of Pediatrics), Takayuki Okamoto

- KKR Sapporo Medical Center (Department of Pathology), Akira Suzuki

Tohoku District
- Iwate Prefectural Central Hospital (Department of Nephrology and Rheumatology), Jun Soma, Izaya Nakaya, Noriyo Sasaki
- Fukushima Medical University School of Medicine (Department of Nephrology, Hypertension, Diabetology, Endocrinology, and Metabolism), Masaaki Nakayama, Koichi Asahi, Hiroaki Satoh
- Japan Community Health care Organization Sendai Hospital (Department of Nephrology), Toshinobu Sato, Asako Fujimori, Satoru Sanada, Mitsuhiro Sato
- Tohoku University Hospital and affiliated hospitals (Internal Medicine), Hiroshi Sato, Mariko Miyazaki, Takashi Nakamichi, Tae Yamamoto
- Yamagata University School of Medicine (Department of Cardiology, Pulmonology, and Nephrology), Tsuneo Konta, Kazunobu Ichikawa
- Yamagata University School of Medicine (Department of Pediatrics), Daisuke Ogino

Kanto District
- National Hospital Organization Chiba-East Hospital (Department of Pathology), Hiroshi Kitamura, (Department of Internal Medicine),Toshiyuki Imazawa, (Department of Pediatrics), Chieko Matsumura,(Department of Surgery), Naotake Akutsu, (Department of Urology), Koichi Kamura
- Dokkyo Medical University Koshigaya Hospital (Department of Nephrology), Tetsuro Takeda
- Dokkyo Medical University (Department of Cardiology and Nephrology), Toshihiko Ishimitsu
- Gunma University Graduate School of Medicine (Department of Nephrology and Rheumatology), Keiju Hiromura
- Jichi Medical University (Division of Nephrology), Daisuke Nagata, Shigeaki Muto, Osamu Saito, Tetsu Akimoto
- The Jikei University School of Medicine (Division of Nephrology and Hypertension), Takashi Yokoo, Nobuo Tsuboi, Kentaro Koike
- The Jikei University School of Medicine, Katsushika Medical Center (Division of Nephrology and Hypertension), Masato Ikeda, Shinya Yokote
- The Jikei University School of Medicine, Daisan Hospital (Division of Nephrology and Hypertension), Kazushige Hanaoka, Hiroyuki Ueda, Junichiro Kato, Mai Tanaka
- The Jikei University Kashiwa Hospital (Division of Nephrology and Hypertension), Makoto Ogura, Akihiko Hamaguchi, Hideyuki Itoh
- Juntendo University Faculty of Medicine (Department of Nephrology), Yusuke Suzuki, Miyuki

Takagi, Hitoshi Suzuki, Teruo Hidaka

- Kawaguchi Municipal Medical Center (Division of Nephrology 1), Masahiro Ishikawa
- Kyorin University School of Medicine (Department of Urology), Kikuo Nutahara
- Kyorin University School of Medicine (Division of Nephrology and Rheumatology, First Department of Internal Medicine), Yoshihiro Arimura, Shinya Kaname
- Mito Saiseikai General Hospital (Division of Nephrology), Itaru Ebihara, Chihiro Satho
- Nippon Medical School (Division of Nephrology, Department of Internal Medicine), Shuichi Tsuruoka, Yukinao Sakai, Akio Hirama
- Nihon University School of Medicine (Division of Nephrology, Hypertension and Endocrinology), Yoshinobu Fuke
- Saitama Medical University, Faculty of Medicine (Department of Nephrology), Hirokazu Okada, Tsutomu Inoue
- Saitama Medical University, Saitama Medical Center (Department of Nephrology and Hypertension), Takatsugu Iwashita, Yuuta Kogure, Kouichi Kanouzawa, Hajime Hasegawa
- Showa University School of Medicine (Division of Nephrology, Department of Medicine), Takanori Shibata
- Showa University Fujigaoka Hospital (Division of Nephrology, Department of Medicine), Yoshihiko Inoue
- St. Marianna University School of Medicine (Division of Nephrology and Hypertension, Department of Internal Medicine), Daisuke Ichikawa, Sayuri Shirai, Yugo Shibagaki
- Tokai University School of Medicine (Division of Nephrology, Endocrinology and Metabolism), Takehiko Wada, Masafumi Fukagawa
- Teikyo University School of Medicine (Department of Internal Medicine), Yoshihide Fujigaki
- Teikyo University School of Medicine (Department of Urology), Shigeo Horie, Satoru Muto
- Tokyo Medical University Ibaraki Medical Center (Department of Nephrology), Masaki Kobayashi, Kouichi Hirayama, Homare Shimohata
- Tokyo Metropolitan Children's Medical Center (Department of General Pediatrics), Hiroshi Hataya
- Tokyo Women's Medical University (Department of Pediatric Nephrology), Motoshi Hattori, Kiyonobu Ishizuka, Noriko Sugawara
- Tokyo Women's Medical University (The Forth Department of Medicine), Kosaku Nitta, Keiko Uchida, Takahito Moriyama
- Toranomon Hospital, Nephrology Center, Yoshifumi Ubara, Tatsuya Suwabe, Junichi Hoshino, Noriko Hayami
- The University of Tokyo (Department of Nephrology and Endocrinology), Masaomi Nangaku, Tetsuhiro Tanaka, Yoshifumi Hamasaki, Kenjiro Honda

- University of Tokyo（Department of Pediatrics), Yutaka Harita, Kenichiro Miura
- University of Tsukuba (Department of Nephrology), Kunihiro Yamagata, Joichi Usui, Tetsuya Kawamura
- Yokohama City University Graduate School of Medicine (Department of Medical Science and Cardiorenal Medicine), Kouichi Tamura, Junji Yamauchi
- Yokohama City University Medical Center, Nobuhito Hirawa, Sanae Saka, Akira Fujiwara

Koushinetsu District

- Niigata University Graduate School of Medical and Dental Sciences (Division of Clinical Nephrology and Rheumatology), Ichiei Narita, Shin Goto, Yumi Itoh, Naofumi Imai
- Shinshu University School of Medicine (Division of Nephrology), Yuji Kamijo, Koji Hashimoto, Akinori Yamaguchi, Yosuke Yamada
- University of Yamanashi Hospital (Third Department of Internal Medicine), Fumihiko Furuya, Daiichiro Akiyama, Kazuya Takahashi, Kenichiro Kitamura

Hokuriku District

- National Hospital Organization Kanazawa Medical Center (Department of Nephrology and Rheumatology), Kiyoki Kitagawa
- Kanazawa Medical University School of Medicine (Department of Nephrology), Hitoshi Yokoyama, Keiji Fujimoto, Norifumi Hayashi
- Kanazawa Medical University (Department of Diabetology & Endocrinology), Daisuke Koya, Yuka Kurosima,
- Kanazawa University Hospital (Division of Nephrology), Takashi Wada, Kengo Furuichi, Miho Shimizu, Norihiko Sakai
- Komatsu Sophia Hospital, Yasuhiro Katou, Taito Miyake
- Moriyama Koshino Clinic, Yoshitaka Koshino
- Pubulic Central Hospital of Matto-Ishikawa, Kazuya Takasawa, Chikako Takaeda
- Sugita Genpaku Memorial Obama Municipal Hospital, Haruyoshi Yoshida, Takayasu Horiguchi
- Toyama Prefectural Central Hospital (Department of Internal Medicine), Masahiko Kawabata
- Toyama City Hospital (Department of Internal Medicine), Satoshi Ota, Yoh-ichi Ishida
- University of Fukui, Faculty of Medical Sciences (Division of Nephrology, Department of General Medicine), Masayuki Iwano, Hideki Kimura, Naoki Takahashi, Kenji Kasuno
- University of Toyama (Second Department of Internal Medicine), Hidenori Yamazaki

Tokai District

- Aichi Children's Health and Medical Center (Department of Pediatric Nephrology), Naoya Fujita, Satoshi Yamakawa
- Aichi Medical University School of Medicine (Division of Nephrology and Rheumatology), Keisuke Suzuki, Takuhito Nagai, Naoto Miura, Hirokazu Imai
- Chuno Kosei Hospital, Shogo Kimura, Yuka Soga
- Fujinomiya City General Hospital, Masanori Sakakima, Kazuto Kitajima, Taichi Sato, Yutaro Kawakatsu
- Fujita Health University School of Medicine (Department of Nephrology), Yukio Yuzawa, Hiroki Hayashi, Kazuo Takahashi
- Hamamatsu University School of Medicine, University Hospital (Internal Medicine1, Division of Nephrology), Hideo Yasuda, Naro Ohashi, Takamasa Iwakura
- Japanese Red Cross Nagoya Daini Hospital (Kidney Center), Asami Takeda, Yasuhiro Otsuka
- Nagoya City East Medical Center, Minamo Ono, Tatsuya Tomonari
- Nagoya City University Graduate School of Medical Sciences (Department of Cardio-Renal Medicine and Hypertension), Michio Fukuda, Masashi Mizuno, Yoshiaki Ogiyama, Ryo Sato
- Nagoya Kyoritsu Hospital (Department of Internal Medicine), Hirotake Kasuga
- Nagoya University Graduate School of Medicine (Department of Nephrology), Seiichi Matsuo, Shoichi Maruyama, Yoshinari Yasuda
- Shizuoka General Hospital (Department of Nephrology), Noriko Mori, Satoshi Tanaka
- Mie University Graduate School of Medicine (Department of Cardiology and Nephrology), Eiji Ishikawa, Mika Fujimoto, Tomohiro Murata, Masaaki Ito
- Yokkaichi Social Insurance Hospital (Division of Nephrology and Blood Purification), Yasuhide Mizutani, Hitoshi Kodera, Masato Miyake

Kinki District

- Hyogo Prefectural Nisihinomiya Hospital (Department of Pathology), Kazumasa Oka
- Ikeda City Hospital (Division of Nephrology), Nobuyuki Kajiwara
- Kitano Hospital, The Tazukekofukai Medical Research Institute (Division of Nephrology and Dialysis), Tatsuo Tsukamoto, Tomomi Endo, Eri Muso
- Kobe University Graduate School of Medicine (Division of Nephrology and Kidney Center), Shinichi Nishi, Shunske Goto
- Kobe University Graduate School of Medicine (Department of Pediatrics), Kazumoto Iijima, Hiroshi Kaito, Takeshi Ninchoji
- JCHO Kobe Central Hospital, Yoko Adachi
- National Hospital Organization Kyoto Medical Center (Division of Nephrology), Koichi Seta, Kensei Yahata

- Kyoto Prefectural University of Medicine Graduate School of Medical Science (Department of Nephrology), Keiichi Tamagaki, Tetsuro Kusaba, Yayoi Shiotsu
- Kyoto University Graduate School of Medicine (Department of Nephrology), Motoko Yanagita, Hideki Yokoi, Kaoru Sakai, Akira Ishii
- Nara Medical University (First Department of Internal Medicine), Yoshihiko Saito, Kenichi Samejima
- National Cerebral and Cardiovascular Center (Division of Hypertension and Nephrology), Satoko Nakamura
- Osaka City University Graduate School of Medicine (Department of Nephrology), Eiji Ishimura, Katsuhito Mori, Akihiro Tsuda, Shinya Nakatani
- Osaka City General Hospital (Division of Nephrology and Hypertension),Yoshio Konishi, Takashi Morikawa, Chizuko Kitabayashi
- Osaka General Medical Center (Department of Kidney Disease and Hypertension), Terumasa Hayashi, Tatsuya Shoji
- Osaka Medical Center and Research Institute for Maternal and Child Health (Department of Pediatric Nephrology and Metabolism), Katsusuke Yamamoto
- Osaka Red Cross Hospital (Department of Nephrology), Akira Sugawara, Masao Koshikawa, Yoshihisa Ogawa, Tomoko Kawanishi
- Osaka University Graduate School of Medicine (Department of Nephrology), Yoshitaka Isaka, Ryohei Yamamoto, Tomoko Namba
- Osaka Medical College (Department of Pediatrics), Akira Ashida
- Saiseikai Shiga Hospital (Division of Nephrology), Toshiki Nishio
- Shiga University of Medical Science (Department of Medicine), Shinichi Araki
- Shirasagi Hospital (Kidney Center), Shigeichi Shoji, Kenjiro Yamakawa, Senji Okuno
- Toyonaka Municipal Hospital (Division of Nephrology), Megumu Fukunaga
- Wakayama Medical University (Department of Pediatrics), Yuko Shima
- Wakayama Medical University (Department of Nephrology), Takashi Shigematsu, Masaki Ohya

Chugoku District

- Kawasaki Medical School (Department of Nephrology and Hypertension), Naoki Kashihara, Tamaki Sasaki, Sohachi Fujimoto
- Kurashiki Central Hospital (Division of Nephrology), Kenichiro Asano, Motoko Kanzaki
- Hiroshima University Hospital (Department of Nephrology), Takao Masaki, Shigehiro Doi, Ayumu Nakashima, Toshinori Ueno
- Mizushima Kyodo Hospital (Department of Nephrology), Nobuyoshi Sugiyama, Yuichirou

Inaba, Kan Yamazaki, Kouji Ozeki

- Okayama Saiseikai General Hospital (Department of Nephrology), Makoto Hiramatsu, Keisuke Maruyama, Noriya Momoki
- Okayama University Graduate School of Medicine, Dentistry, and Pharmaceutical Sciences (Department of Medicine and Clinical Science), Tatsuyuki Inoue, Keiko Tanaka, Ayu Akiyama
- Saiseikai Yamaguchi General Hospital (Department of Internal Medicine), Tsuyoshi Imai
- Shimane University Faculty of Medicine (Division of Nephrology), Takafumi Ito
- Tottori University, Faculty of Medicine (Division of Pediatrics and Perinatology), Shinichi Okada, Koichi Kitamoto, Hiroki Yokoyama, Yuko Yamada

Shikoku District

- Kagawa University, Faculty of Medicine (Department of Cardiorenal and Cerebrovascular Medicine & Department of Clinical Pathology), Tetsuo Minamino, Tadashi Sofue, Yoko Nishijima, Yoshio Kushida
- Kochi University, Kochi Medical School (Department of Endocrinology, Metabolism and Nephrology), Yoshio Terada, Taro Horino, Yoshinori Taniguchi, Yoshiko Shimamura
- Kochi University, Kochi Medical School (Department of Pediatrics), Mikiya Fujieda, Masayuki Ishihara
- Tokushima University Graduate School (Department of Pediatrics, Institute of Biomedical Sciences), Shoji Kagami, Maki Urushihara, Yukiko Kinoshita
- Tokushima University Graduate School (Department of Nephrology, Institute of Biomedical Sciences), Toshio Doi, Hideharu Abe, Kojiro Nagai

Kyushu District

- Fukuoka University (Division of Nephrology and Rheumatology, Department of Internal Medicine, Faculty of Medicine), Hitoshi Nakashima, Yoshie Sasatomi, Satoshi Hisano
- Japanese Red Cross Fukuoka Hospital (Department of Pediatrics), Ken Hatae, Maiko Hinokiyama, Rie Kuroki, Hiroyo Maruyama
- Japanese Red Cross Fukuoka Hospital (Nephrology and Dialysis Center), Koji Mitsuiki
- Kumamoto University Graduate School of Medical Sciences (Department of Nephrology), Masashi Mukoyama, Masataka Adachi
- Kurume University School of Medicine (Division of Nephrology, Department of Medicine), Kei Fukami, Jyunko Yano
- Kyushu University Graduate School of Medical Sciences (Department of Medicine and Clinical Science), Kazuhiko Tsuruya, Shunsuke Yamada, Akihiro Tsuchimoto, Hisako Yoshida

- Kyushu University Graduate School of Medical Sciences (Department of Environmental Medicine), Yutaka Kiyohara, Toshiharu Ninomiya, Masaharu Nagata
- Miyazaki Prefectural Miyazaki Hospital (Division of Nephrology), Naoko Yokota-Ikeda, Shigehiro Uezono, Keiko Kodama
- Nagasaki University Hospital (Department of Pathology), late Takashi Taguchi
- Nagasaki University Hospital (Department of Nephrology), Tomoya Nishino, Hideyuki Arai, Yoko Obata, Tadashi Uramatsu
- National Fukuoka Higashi Medical Center (Kidney Unit), Ritsuko Katafuchi
- National Hospital Organization Kyushu Medical Center, Masaru Nakayama
- Oitaken Kouseiren Tsurumi Hospital (Division of Nephrology), Ryokichi Yasumori
- Saga University, Faculty of Medicine (Department of Internal Medicine), Yuji Ikeda, Motoaki Miyazono, Makoto Fukuda, Tsuyoshi Takashima
- St. Mary's Hospital, Harumichi Higashi
- University of Miyazaki Hospital (First Department of Internal Medicine), Shouichi Fujimoto, Yuji Sato, Masao Kikuchi
- University of Occupational and Environmental Health (Second Department of Internal Medicine), Masahito Tamura,Tetsu Miyamoto
- University of the Ryukyus Graduate School of Medicine (Department of Cardiology, Nephrology and Neurology), Yusuke Ohya, Kentaro Kohagura
